# Ocular Complications Following Autologous Fat Injections into Facial Area: Case Report of a Recovery from Visual Loss After Ophthalmic Artery Occlusion and a Review of the Literature

**DOI:** 10.1007/s00266-017-0805-3

**Published:** 2017-02-23

**Authors:** Aleksandra Szantyr, Michał Orski, Ida Marchewka, Mariusz Szuta, Małgorzata Orska, Jan Zapała

**Affiliations:** 1 Department of Cranio-Maxillo-Facial, Oncological and Reconstructive Surgery, Jagiellonian University Medical College, Ludwik Rydygier Memorial Specialized Hospital, 31-826 Kraków, Os. Złotej Jesieni 1, Cracow, Poland; 2 Department of Ophthalmology, Ludwik Rydygier Memorial Specialized Hospital, Cracow, Poland

**Keywords:** Vision loss, Blindness, Facial fillers, Filler injection, Autologous fat

## Abstract

With the increase in popularity of the use of cosmetic fillers in plastic and esthetic surgery, the possibility of severe ocular complications should not be neglected. Of the fillers used, autologous fat is the most common to cause permanent visual deterioration, one of the most severe complications associated with the use of cosmetic fillers. Here we present the first report of a complete recovery of visual acuity from an instance of visual loss with no light perception caused by ophthalmic artery occlusion of the right eye following autologous fat injection in the facial area. Immediate ophthalmological intervention and comprehensive therapy with prostaglandins and vinpocetine made it possible to restore retinal perfusion and achieve complete recovery of visual acuity. Awareness of the iatrogenic artery occlusions associated with facial fillers and the need for immediate treatment should be popularized among injectors to prevent devastating consequences, such as permanent vision loss.

*Level of Evidence V* This journal requires that authors assign a level of evidence to each article. For a full description of these Evidence-Based Medicine ratings, please refer to the Table of Contents or the online Instructions to Authors www.springer.com/00266.

## Introduction

With the increase in popularity of the use of cosmetic fillers in plastic and esthetic surgery, the possibility of severe ocular complications should not be neglected. Permanent visual deterioration, one of the most severe complications associated with the use of cosmetic fillers, can be the result of ophthalmic artery occlusion (OAO), generalized posterior ciliary artery occlusion (generalized PCAO) with relative sparing of the central retinal artery, central retinal artery occlusion (CRAO), localized PCAO, branch retinal artery occlusion (BRAO) and posterior ischemic optic neuropathy (PION). Another classification includes diffuse occlusions (OAO, generalized PCAO, CRAO) and localized occlusions (localized PCAO, BRAO, PION) [1, 2]. Autologous fat injections are associated with diffuse occlusions, such as OAO [1] and central retinal artery occlusion [3], and are therefore characterized by more severe clinical symptoms and poorer visual prognosis when compared to other facial fillers. The proposed underlying mechanism assumes intravascular injection and retrograde embolization of the filler, as the injected bolus may overcome arterial pressure and move against the direction of blood flow [3, 4]. When the injector releases the plunger, the filler travels with blood flow and enters the ophthalmic artery and its branches. With higher injection pressures, filler particles may be pushed further retrograde and enter the brain circulation, which in turn results in cerebral infarction. Autologous fat is the most common filler type to cause iatrogenic blindness, being responsible for almost 48% of the cases of cosmetic filler-induced visual loss reported in the literature up to the present [3]. Ocular complications occur most frequently after injection to the glabellar area, nasal region, nasolabial fold and forehead. However, due to the complex vascular anatomy of the face, essentially any location of the facial region may be at risk of ocular complications. Ocular symptoms most commonly include: sudden unilateral vision loss, ocular pain and headache, which are most likely to occur immediately after injection. Visual loss may be accompanied by ophthalmoplegia and ptosis [2, 3, 5, 6]. Although recovery from ophthalmoplegia and ptosis is observed in the majority of cases, vision loss is usually permanent. From 47 cases of ocular complications following autologous fat injections reported in the English language literature up to 2015, 38 cases resulted in complete vision loss [5]. In 5 cases, some remaining visual acuity was observed, varying from light perception to 20/40 at follow-up, but no improvement was observed in any of the reported cases, despite the application of various management strategies [5]. Although several treatment options have been proposed and reported [1, 2, 5, 6], until now no treatment has been proved to be effective. As results are usually unsatisfactory, there is little to no evidence for improvement with any proposed treatment. Therefore, we present here a report of a case of visual acuity recovery from complete visual loss with no light perception caused by ophthalmic artery occlusion of the right eye following autologous fat injection in the facial area.

## Case Presentation

A 38-year-old male with a posttraumatic defect of the squamous part of the forehead bone underwent soft tissue augmentation with autologous fat injection in his left supraorbital and right forehead area. Two years earlier, the patient had been hit by a piece of wood during work and suffered a bilateral fracture of the anterior cranial fossa and bilateral naso-orbital fracture. He underwent microplate osteosynthesis of the naso-orbital fractures as well as repair of the dura mater of the anterior cranial fossa. Eighteen months after the accident, the patient received his first autologous fat injection into his right forehead and supraorbital area with no intraoperative or postoperative complications. The patient was content with the esthetic results of the first procedure and wanted a second one to improve the results. The procedure was performed under local anesthesia (lidocaine with norepinephrine) with Byron Medical instrumentation. After infiltration of the donor’s abdominal area with the LAMIS™ Infiltration Cannula (14 ga outer diameter, 30 cm length), autologous fat was harvested with COLEMAN™ Aspiration Cannula (11 ga outer diameter, 15 cm length) using the lipoaspiration technique and prepared with the standard procedure. Fat was subcutaneously injected with the COLEMAN™ Style Mini Infiltration Cannula (20 ga outer diameter, 3.175 cm length) and a 2-ml injecting syringe. Through two small incisions in the right forehead and supraorbital area, 5 ml of fat was injected into the left supraorbital area and 5 ml into the right forehead area. No scar release maneuvers were performed. During the injection of the final 0.5 ml of fat into the right forehead area, the patient reported ocular pain and flashes of light in front of the right eye, followed by a complete visual loss with no light perception in the right eye. Since occlusion of the right ophthalmic artery was suspected, the patient was immediately transferred to the Ophthalmology Ward. On examination, no light perception and a relative afferent pupillary defect in the right eye were observed. Fundus examination of the right eye showed pale, diffused edema of the retina and severely narrowed retinal arterioles with poor perfusion. Multiple shiny particles were observed within the lumen of the arteries, consistent with fat embolism. Ophthalmic artery occlusion of the right eye was diagnosed. Treatment was administered immediately. The time from the first symptoms to the beginning of treatment was less than 20 min. The administered treatment included prolonged circular digital and contact lens-induced ocular massage, ocular drops: 0.5% timolol, brimonidine and dorzolamide to the right eye, 24 mg dexamethasone intravenously, 500 ml of 20% mannitol intravenously, 80 ml of 40% glycerol per os, 500 mg of acetazolamide per os. Computed head tomography with contrast did not reveal any new pathologies apart from the preexisting fluid hypodense areas localized in the right frontal lobe with the partial defect of the squamous part of the forehead bone caused by the prior trauma. Slow visual recovery was observed within 90 min. Examination of the fundus of the right eye performed after 150 min from the first symptoms revealed patchy areas of retinal edema and partially perfused retinal arteries. At that time, the patient reported multiple irregular visual field deficits. Further treatment included alprostadil (Prostavasin) 40 mcg administered intravenously (with the mean arterial pressure assessment every 15 min during the intravenous infusion) once a day for 6 days, dexamethasone intravenously in decreasing dosages scheme (2 × 8 mg on the second day, 3 × 4 mg on the third day, 3 × 2 mg on the fourth day).

The patient was discharged on the sixth day after the visual loss in good local and general condition. The best corrected visual acuity in the right eye improved to 0.1 logMAR. The standard automated perimetry showed irregular visual field deficits, and optical coherence tomography (OCT) revealed significant damage to the ganglion cell complex (GCC). Pharmacotherapy, with oral 5 mg vinpocetine daily, was continued after discharge. In the 2-year follow-up, visual acuity has remained stable, visual field examination has shown no visual deficits, and OCT has shown no further decrease in GCC.

## Discussion

Visual loss is one of the most severe complications reported in patients undergoing facial filler injections. In the majority of reported cases, visual deterioration was severe and irreversible [2, 5, 7]. Autologous fat injections are associated with the most severe visual impairment [2, 5, 8, 9]. Due to differences in particle size among the different types of facial fillers, patients with ocular symptoms following other types of fillers, e.g., hyaluronic acid injections, are more likely to suffer from localized ocular occlusion with milder clinical manifestations and better visual prognosis than patients who received autologous fat injections [5, 8–10]. Preventative strategies proposed in the literature to reduce the risk of vascular complications following facial filler injections include: using small-diameter blunt flexible cannulas or non-traumatic blunt tip needles instead of sharp cannulas and needles, limiting syringe size to 1 ml, aspirating before injection, injecting the filler slowly with minimal pressure, limiting the amount of filler to less than 0.1 ml with each pass, mixing the filler with vasoconstrictor, using a topical vasoconstrictor before injection and moving the needle tip while injecting [5, 7, 11, 12]. Avoiding injection at the sites of previous trauma, or at sites with chronic inflammation or scarring, is also advised. Facial filler injections in patients who have previously undergone facial or plastic surgeries should be performed with extra caution [5–7].

According to the literature, no fully effective treatment is available. Nevertheless, there are several papers presenting various treatment modalities that recover some degree of visual function. The aim of the treatment after facial filler embolism is to restore the perfusion of the retina as soon as possible. The retina is very sensitive to hypoxia, and after 90 min damage due to retinal ischemia becomes permanent [1, 3, 5, 7, 13]. Most previously reported cases of vision loss secondary to fat embolism have remained without any improvement. Therefore, no evidence-based treatment strategies are available. Slightly better outcomes have been reported in cases of vision loss following injection of fillers other than autologous fat [5, 7]. From 23 cases of ocular complications following injections of hyaluronic acid into the facial area, permanent vision loss was observed in 9 cases, and in 6 cases some level of improvement in visual acuity was reported [5]. Treatment is aimed at lowering intraocular pressure to boost retinal perfusion, dilating arteries to eliminate embolus and reducing the inflammatory component of the injury. To decrease intraocular pressure, eye drops are administered (e.g., alpha agonists, B-blockers, carbonic anhydrase inhibitors) and ocular massage and anterior chamber paracentesis are performed. Carbon dioxide and oxygen inhalation or vasodilatory agents such as prostaglandin E1 provoke arterial dilation. Hyperbaric oxygen therapy and systemic corticosteroids decrease the inflammatory response. [1–3, 5, 6, 13]. Table 1 presents the currently recommended treatments and their underlying mechanism of action.

**Table 1:** Currently recommended treatments for vision loss following autologous fat injections into facial area

Treatment	Mechanism of action
Timolol 0.5% drop administered topically	Lowers intraocular pressure and dislodges the embolus to a more peripheral downstream location [5, 7, 9]
Acetazolamide 500 mg per os or intravenously	Reduces intraocular pressure that may increase blood flow in the retina [7]
Nitroglycerin 2% paste or sublingual isosorbide dinitrate or systemic pentoxifylline	Dilates the retinal arteries [7, 14]
Intravenous infusion of mannitol 20% (100 ml over 30 min)	Lowers intraocular pressure and dislodges the embolus to a more peripheral downstream location [7, 9]
Ocular massage—performed digitally or using a Goldmann fundus contact lens [9]	Decreases intraocular pressure and increases blood flow in the arterioles, potentially dislodging the embolus [7, 9, 15]
Anterior chamber paracentesis	Rapidly reduces intraocular pressure and encourages blood flow in the retina [5, 7]
Systemic and topical corticosteroids	Decreases retinal edema and inflammatory reaction [6, 7]
Hyperbaric oxygen therapy	Reverses any salvageable retinal damage [6, 9]
Inhalation of carbogen (95% oxygen with 5% carbon dioxide)	Dilates the retinal arteries and increases delivery of oxygen [5, 7]
Intravenous prostaglandin E1	Causes vasodilatation and increases blood flow in the retina, decreases activation of thrombocytes, improves cell metabolism by increasing oxygenation, decreases activation of neutrophils and the release of their toxic metabolites, helping to reduce tissue damage from inflammation and possibly from hypoxia [5–7]
Anticoagulation with oral acetylsalicylic acid or low molecular weight heparin	Prevents further thrombosis [7, 9]

The crucial factor in the management strategy is the immediate assessment and referral to the specialist in the nearby area, preferably an ophthalmologist or retina specialist, to start the proper treatment within the 90-min time span after which the damage to the retina is irreversible [10, 13]. Loh et al. [13] have proposed a treatment algorithm for managing vision loss following filler injections. When a patient presents the first symptoms of vascular compromise in the retina, filler injection should be instantly stopped and the patient should be laid in a supine position. Immediate treatment (including topical instillation of timolol, and/or oral acetazolamide and ocular massage) should be administered by a non-ophthalmologist injector before arranging and transferring the patient to a specialist for a definitive therapy, which consists of further medical administration and—when indicated—anterior chamber paracentesis. In cases of hyaluronic acid-induced vision loss, hyaluronidase may be used to dissolve hyaluronic acid emboli [1–14]. After these acute measures have been taken, additional supportive therapy (corticosteroids, hyperbaric oxygen therapy, anticoagulants) should be introduced to protect retinal cells.

In our case, treatment was started within 20 min of the first symptoms of vision loss. The successful outcome can also be ascribed to the comprehensiveness of the treatment aimed at restoring perfusion of the retina with the use of multiple drugs and management strategies. In previously reported cases, the treatment provided has usually been incomplete, since the majority of patients suffering from ocular complications received at most a 3-step therapy [6, 7]. As noted by Chen et al. [7], combination therapy may contribute to recovery of visual symptoms. In our case, in addition to commonly used treatment options such as ocular massage, acetazolamide, mannitol and corticosteroids, we also used alprostadil and vinpocetine. Alprostadil is a synthetic variant of prostaglandin E1. It causes vasodilatation through directly affecting the vascular smooth muscles and increases blood flow in the retina. Alprostadil also decreases thrombocyte activation, improves cell metabolism by increasing oxygen supply to the tissues and decreases neutrophil activation and the release of their toxic metabolites, helping to reduce tissue damage caused by inflammation and possibly by hypoxia. The use of prostaglandin E1 as a part of comprehensive therapy for visual loss following hyaluronic acid injection was also reported by Chen et al. [7] and resulted in improvement in visual acuity, extraocular movement and visual field defects. Vinpocetine, chemically known as ethyl apovincamine, is a vinca alkaloid that increases cerebral blood flow and has neuroprotective effects [16]. This drug is used in the treatment of ischemic cerebrovascular diseases and vascular dementias. Vinpocetine may help to increase retinal perfusion and prevent further damage to still-viable central retinal tissue.

## Conclusions

The common use of facial injections, most often performed in esthetic medicine, has resulted in an increased number of ocular complications. In the presented case, after autologous fat injection the patient suffered from sudden complete vision loss in one eye. Fundus examination and specific symptoms enabled a diagnosis of ophthalmic artery occlusion. Immediate ophthalmological intervention and comprehensive therapy with prostaglandins and vinpocetine made it possible to restore retinal perfusion and achieve complete recovery of visual acuity. Awareness of iatrogenic artery occlusions associated with facial fillers and the need for immediate treatment should be popularized among injectors to prevent devastating consequences such as permanent vision loss.
